# Antimicrobial Activity from Putative Probiotic Lactic Acid Bacteria for the Biological Control of American and European Foulbrood Diseases

**DOI:** 10.3390/vetsci9050236

**Published:** 2022-05-12

**Authors:** Massimo Iorizzo, Sonia Ganassi, Gianluca Albanese, Francesco Letizia, Bruno Testa, Cosimo Tedino, Sonia Petrarca, Franco Mutinelli, Alessandra Mazzeo, Antonio De Cristofaro

**Affiliations:** 1 Department of Agricultural, Environmental and Food Sciences, University of Molise, 86100 Campobasso, Italy; iorizzo@unimol.it (M.I.); sonia.ganassi@unimol.it (S.G.); f.letizia@studenti.unimol.it (F.L.); bruno.testa@unimol.it (B.T.); cosimoted_91@libero.it (C.T.); sonia_petrarca@libero.it (S.P.); alessandramazzeo@unimol.it (A.M.); decrist@unimol.it (A.D.C.); 2 Conaproa, Consorzio Nazionale Produttori Apistici, 86100 Campobasso, Italy; 3 Istituto Zooprofilattico Sperimentale delle Venezie (IZSVe), National Reference Laboratory for Honey Bee Health, Viale dell’Università 10, 35020 Legnaro, Italy; fmutinelli@izsvenezie.it

**Keywords:** *Apilactobacillus kunkeei*, lactic acid bacteria, *Lactiplantibacillus plantarum*, *Melissococcus plutonius*, *Paenibacillus larvae*, probiotic

## Abstract

The balance of the gut microbiome is important for the honey bee’s growth and development, immune function and defense against pathogens. The use of a beneficial bacteria-based strategy for the prevention and biocontrol of American foulbrood (AFB) and European foulbrood (EFB) diseases in honey bees offers interesting prospects. Lactic acid bacteria (LAB) are common inhabitants of the gastrointestinal tract of the honey bee. Among LABs associated with bee gut microbiota, *Lactiplantibacillus plantarum* (previously *Lactobacillus plantarum*) and *Apilactobacillus kunkeei* (formerly classified as *Lactobacillus kunkeei*) are two of the most abundant species. In this study, four *Lactiplantibacillus plantarum* strains and four *Apilactobacillus kunkeei* strains, isolated from the gastrointestinal tract of honey bee (*Apis mellifera* L.) were selected for their in vitro inhibition ability of *Paenibacillus larvae* ATCC 9545 and *Melissococccus plutonius* ATCC 35311. In addition, these LABs have been characterized through some biochemical and functional characteristics: cell surface properties (hydrophobicity and auto-aggregation), carbohydrates assimilation and enzymatic activities. The antimicrobial, biochemical and cell surface properties of these LABs have been functional to their candidature as potential probiotics in beekeeping and for the biocontrol of AFB and EFB diseases.

## 1. Introduction

*Apis mellifera* L. is an insect of great ecological importance on a worldwide level both for its role as a pollinator of crops, fruit, and wildflowers and for maintaining the biodiversity of numerous ecosystems. Moreover, as a pollinating insect, it holds a high economic value for hive products [1,2]. Whilst global stocks of managed honeybee colonies have apparently been increasing, considerable losses of wild and domestic bees have been reported in many parts of the world [3,4,5,6].

Biotic and abiotic factors that adversely impact bees’ welfare and survival include changes in land use and management intensity, climate change, beekeeper’s management practices, lack of forage (nectar and pollen), the use of pesticides in agriculture, parasites and pathogen infections [7,8,9,10]. The gut microbiome is crucial for the honey bee’s growth and development, immune function and defense against pathogens. Furthermore, a well-balanced microbiota is important for sustaining the health and vigor of honey bees [11]. The gut microbiota can be influenced by the aforementioned factors and its imbalance (dysbiosis) can lead to a weakening of honey bees and contribute to Colony Collapse Disorder (CCD) [11,12,13].

The digestive tract of honey bees is the site of infections triggered by pathogenic agents of brood disease, such as *Paenibacillus larvae* and *Melissococcus plutonius*, which can cause considerable losses in beekeeping and agriculture as they adversely influence the survival of both managed and wild honey bees [14]. *M. plutonius* (Order: *Lactobacillales*; Family: *Enterococcaceae*), a Gram-positive bacterium [15,16] resulting in the outbreak of European foulbrood (EFB), a globally damaging brood disease occurring in *Apis mellifera* L. [17,18]. *P. larvae* (Order: *Bacillales; Family: Paenibacillaceae*), a flagellated, spore-forming Gram-positive bacterium, is the causative agent of quarantine disease American foulbrood (AFB), the most severe and globally distributed brood disease affecting *Apis mellifera* in the larval and pupal stages [19,20,21]. Heavy brood losses, colony collapse and extreme contagiousness render EFB and in particular AFB economically important and notifiable diseases in many world regions [22].

To date, to prevent the further spread of the two pathogens, the destruction of symptomatic colonies and the surveillance of nearby apiaries are the only means to address them [9,23]. However, the beekeeping practice-based management of EFB is considered since this is a conditioned disease, e.g., feeding, adding healthy honey bees, and artificial replacement of the queen [24]. Since it is only effective against the vegetative form of the above-mentioned pathogens, the use of antibiotics for controlling these diseases is an unsustainable strategy. Moreover, long-lasting treatments and their common use in prophylaxis, as well as failing to eliminate bacterial spores, may cause or accelerate mechanisms of antibiotic resistance [25,26,27,28]. Additionally, antibiotics may leave residues in hive products [29,30,31] and also cause honey bee gut community dysbiosis [32,33,34]. On this matter, a growing number of research studies have revealed the relationship between disease states and variation in the native microbiome of honey bees, suggesting that the integrity of the microbiome correlates with disease susceptibility and, on a more general level, with the health status of honey bees [11,31,35,36,37,38,39]. For all these reasons, the use of antibiotics is now tightly regulated and not recommended in most European countries [29,30,31,40]. Further measures to control AFB disease include enzymes (e.g., glucose oxidase) [41]; heterocyclic organic compounds, such as indoles, to inhibit the germination of *P*. *larvae* spores [42]; bacteriophages [43]; and selective breeding for hygienic behaviour [44]. However, these methods are often inefficient or not applicable for prophylactic purposes, leaving honey bee colonies susceptible to AFB [45,46]. The adoption of biological alternatives for disease control could represent a more viable management strategy. Herbal and propolis extracts, as well as spices and essential oils, showed antimicrobial action towards *P. larvae* and *M. plutonius* [46,47,48,49,50]. Nevertheless, their efficacy is often limited by the resilient nature of *P. larvae* spores that can remain infectious for more than 35 years [20]. Recently, research has focused on the intestinal microbial composition of bees as a possible natural alternative for the control of different diseases [51,52]. The structure of the gut bacterial community can be used as an indicator of honey bee health, and maintaining its balance is the key to supporting the health and well-being of honey bees [11,35,53,54,55]. Several studies have shown that the dysbiosis of the gut microbial community can promote the incidence of EFB and AFB diseases [38,40]. Other investigations have reported that the use of probiotics as a supplement in the honey bee diet could improve the health status of bees [36,56,57], counteract bee pathogens and parasites, or enhance bee immunity [51,52,58].

Lactic acid bacteria (LAB) are common inhabitants of the gastrointestinal tract (GIT) of numerous insects and their presence in the honey bee intestinal tract has been consistently reported in the literature [53,59,60]. The importance of LABs is also underscored by their ecological distribution, which is not limited to the gut of the adult bee. They have been isolated from the gut of larvae [61] and the honey stomach of adult bees [62], which is a further relevant microbial niche associated with food storage and liquid transfer (water, nectar, and royal jelly), adjacent to the midgut. Moreover, LABs are also dominant in the hive environment [63]. Given this background, there is an urgent demand for new approaches to developing an innovative and safe management strategy for the biocontrol of such serious diseases. The use of a beneficial bacteria-based procedure for the prevention and biocontrol of pathogenic microorganisms in honey bees offers interesting prospects [36,64]. It is well established that LAB exerts an antagonistic effect against various bee pathogens through the production of antibacterial metabolites, e.g., organic acids and bacteriocins [56,64,65,66]. Recently, this activity has been assessed against *P. larvae* and *M. plutonius* [53,67,68], the causal agents of the AFB and EFB diseases, respectively. Among LABs associated with bee gut microbiota, *Lactiplantibacillus plantarum* (previously *Lactobacillus plantarum*) and *Apilactobacillus kunkeei* (formerly classified as *Lactobacillus kunkeei*) are two of the most abundant species [60]. *A. kunkeei* is a bacterium that colonizes niches rich in fructose and is therefore classified as fructophilic. Fructophilic lactic acid bacteria (FLAB) are a very recently defined group of LAB [69]. Being fructose, their optimal substrate, they can be found in fermented foods, flowers, and fruits. They are also present in the intestine of insects whose diets are rich in fructose [70]. *L. plantarum* is an important and ubiquitous LAB species characterized by extreme versatility [71]. This bacterium is normally detected in a wide variety of niches, including fermented foods, plants, and the gastrointestinal tract (GIT) of mammals, fish, and insects, including honey bees [72,73,74,75,76,77,78]. Various authors have proven that *L. plantarum* and *A. kunkeei* have antagonistic activity against several pathogens, including *P. larvae* and *M. plutonius* [53,64,79,80].

Several studies have highlighted that the use of generic probiotics may not be beneficial to bees, but rather cause alteration of the symbiotic microflora and depress the immune system of honey bees [81,82]. Therefore, the isolation of bacteria that normally colonize the gut of honey bees is highly recommended for the selection of suitable strains that are functional to enhance the welfare of honey bees and increase their resistance to pathogens.

Based on these considerations, in vitro inhibitory activities of four *L. plantarum* and four *A. kunkeei* strains isolated from the gut of the honey bee (*A. mellifera* L.) against *P. larvae* and *M. plutonius* were evaluated. Furthermore, several functional and biochemical characteristics of these LABs were investigated for feasible application as probiotics in bee diets.

## 2. Materials and Methods

### 2.1. Bee Sample Processing

Worker bees (*A. mellifera* subsp. *mellifera*) were sampled in spring 2020 at an apiary located in the Molise region, in south-eastern Italy, housed in queen bee cages and immediately transported to the laboratory. The apiaries were owned by beekeepers belonging to a beekeeping association (CONAPROA, National Consortium of Beekeeping Producers, Campobasso, Italy).

To obtain the social stomach, the midgut, and the ileum from the honey bee samples, these were killed by removing the head; the removal of the head is necessary not only to facilitate the operator in the dissection, but also to avoid contamination of the samples by allochthonous bacteria (of environmental origin).

The insects were then put in a glass Petri dish containing sterile saline (NaCl 0.9%) and dissected at room temperature using a pair of stainless-steel microdissection scissors and tweezers with fine tips (both washed in alcohol and flame-sterilized). To obtain the social stomach, the distal part of the esophagus and near the proventriculus were cut; the midgut was obtained by cutting at the level of the pyloric valve; and the ileum was removed by performing incisions in the initial part of the rectum [60]. Anatomical specimens from five worker bees were placed in tubes containing sterile physiological solutions in order to isolate bacteria.

### 2.2. Bacterial Isolation

Bacterial colonies were isolated from MRS (Oxoid, Milan, Italy) and modified MRS (2% fructose) agar plates incubated at 35 °C under anaerobic conditions. After 48–72 h, for each of the two-culture media, 10% of the total microbial colonies were picked at random and purified by successive streaking onto MRS agar plates. Gram-positive and catalase-negative strains were selected as presumptive LABs and subsequently characterized.

### 2.3. Genotypic Analysis

Genomic DNA extraction from pure bacterial cultures and PCR amplification was accomplished as described by Iorizzo et al. [77]. Molecular identification of the bacteria was carried out based on 16S rRNA nucleotide sequencing. The 16S rRNA gene region of the genomic DNA was amplified using the universal primers 27F (5′-AGAGTTTGATCCTGGCTCAG-3′) and 1492R (5′-TACGGTTACCTTGTTACGACTT-3′). Sequencing of purified amplified products was analysed using the Basic Local Alignment Search Tool [83] in the NCBI (National Center for Biotechnology Information) database [84]. The phylogenetic analysis of the 65 16S rRNA sequences was performed with MEGA X software [85], using *A. kunkeei* ATCC 700308 and *L. plantarum* ATCC 14917 as reference strains.

### 2.4. Screening of Antimicrobial Activity

A preliminary screening of the antimicrobial activity of sixty-five LABs against *P. larvae* ATCC 9545 and *M. plutonius* ATCC 35311 was carried out by spotting 10 μL overnight LAB cultures (10^8^ CFU/mL) onto the surface of MRS (Oxoid Ltd., Hampshire, UK) agar plates, which were then anaerobically incubated at 35 °C for 24 h to allow colonies development. The pathogens were cultured in 10 mL of *Brain Heart Infusion* (BHI-Oxoid Ltd., Hampshire, UK) at 35 °C for 16 h. Subsequently, 100 μL of overnight culture (10^7^ CFU/mL) were inoculated into 7 mL of BHI soft agar (0.7% agar), maintained at 45 °C and poured over the MRS plates. After incubation at 35 °C for 48 h, the diameter of the clear zone around the LAB colonies, resulting from a lack of pathogen growth, was measured with a caliber in millimeters and expressed as the zone of inhibition (ZOI). The experiment was conducted in triplicate and activity was reported as the diameter of the ZOI ± SD. The LAB strains that showed the greatest antagonistic activity against the two pathogens (ZOI > 4 mm) were used for subsequent tests (Table S1 Supplementary Materials).

### 2.5. Antimicrobial Activity Test

LAB strains (*Lactiplantibacillus plantarum:* LP 31, LP 42, LP 148 and LP 179; *Apilactobacillus kunkeei:* ALK 181, ALK 222, ALK 268 and ALK 385) were grown in MRS broth for 16 h at 35 °C, reaching a cell density of 10^8^ CFU/mL. The bacterial cultures were centrifugated (8000 rpm for 15 min at 4 °C) to separate the cell pellet from the cell-free supernatant (CFS). The supernatant (CFS) was sterilized by filtration (cellulose acetate membrane, pore size 0.22 μm, Sigma-Aldrich; St. Louis, MO, USA) and used for subsequent tests. The antimicrobial activity was tested following Iorizzo et al. [86] protocol and its intensity has been expressed as ZOI [87]. The tests were conducted in triplicate.

### 2.6. Biochemical Characterization

LABs possessing antimicrobial activity against *P. larvae* and *M. plutonius* were screened for their carbohydrate fermentation and enzymatic patterns, using the API 50CHL and the API ZYM system kit (bioMérieux SA, Marcy l’Etoile, France), respectively.

Prior to API ZYM use, the LABs were grown in MRS broth at 35 °C and after 18 h, the cultures were centrifuged, washed and resuspended in sterile saline. The bacterial suspension (BS) was adjusted to a 6 on the McFarland turbidity scale and used for the subsequent phases according to the manufacturer’s instructions. Positive evidence of 19 enzymatic activities was found as a result of suspension chromatic change.

For the application of the API 50CHL, the BS was adjusted to 2 on the McFarland scale and used for the subsequent analysis. The ability of 49 different carbohydrates to be assimilated caused the pH to decrease and generated a colorimetric change in the medium, the composition of which is shown in Table S2 (Supplementary Materials).

### 2.7. Cell Surface Properties

#### 2.7.1. Bacterial Cultures

The LAB strains were grown in MRS broth at 35 °C. After 12 h, the cultures were centrifugated (8000 rpm for 15 min at 4 °C). Subsequently, the bacterial cells were washed three times with physiological solution (NaCl 0.9%) and resuspended in the same solution to an optical density of 0.5 on the MacFarland scale (OD_580_), to standardize the bacterial density at 10^8^ CFU/mL. The OD_580_ of the BS was measured using a spectrophotometer (Multilabel Counter—PerkinElmer 1420, San Jose, CA, USA). The subsequent tests were conducted in triplicate and the measurements were carried out in duplicate.

#### 2.7.2. Auto-Aggregation

The evaluation of the auto-aggregation (AA) capacity was performed according to Cozzolino et al. [88]. Briefly, the BSs were incubated at 35 °C and their OD_580_ was measured using a spectrophotometer (PerkinElmer 1420 Multilabel Counter) after 1, 2, 5 and 24 h. The percentage of AA (AA%) was calculated using the following formula: AA% = [1 − (OD_t_/OD_0_)] × 100, where OD_0_ is the absorbance at time 0 and OD_t_ is the absorbance detected after 1, 2, 5 and 24 h.

#### 2.7.3. Hydrophobicity

The determination of cell surface hydrophobicity was evaluated on LAB strains based on the bacterial ability to adhere to hydrocarbons (BATH), as described by Iorizzo et al. [66], using xylene and toluene. Every single organic solvent was added (1:1 *v/v*) to the BSs and mixed (vortex-type mixer) for 5 min. After 15, 30, and 60 min of incubation at room temperature, the aqueous phase was carefully removed and the values of absorbance were detected at 580 nm using a spectrophotometer (PerkinElmer 1420 Multilabel Counter). Hydrophobicity was expressed as the percentage decrease in the optical density using the following formula: H% = [1 − (OD_0_/OD_t_)] × 100, where OD_t_ represents the absorbance value after the addition of xylene or toluene (15, 30 and 60 min), while OD_0_ represents the absorbance value before the addition of the hydrocarbons.

#### 2.7.4. Statistical Analysis

All the data obtained from the three independent experiments are expressed as mean ± standard deviation (SD). Statistical analysis was performed using an analysis of variance (ANOVA). The obtained data, normally distributed, were analysed using Tukey post hoc tests with ANOVA. Statistical significance was attributed to *p*-values < 0.05. The software SPSS (IBM SPSS Statistics 21) was used for the analysis. 

## 3. Results

### 3.1. LAB Species Diversity 

In total, sixty-five Gram-positive and catalase-negative bacterial strains were assumed to be LABs. 

**Figure 1:**
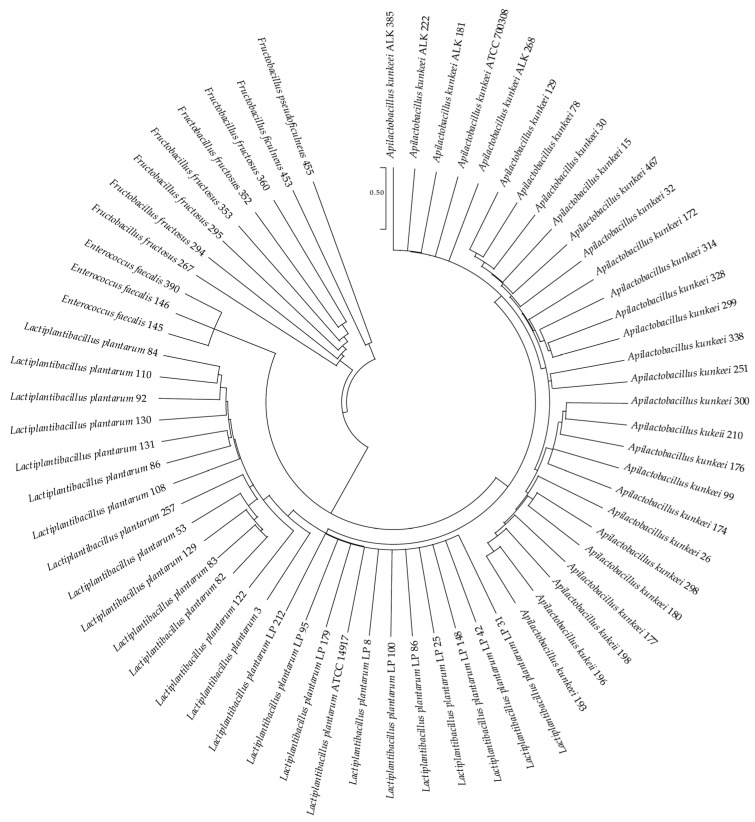
Phylogenetic analysis of the 16S rRNA gene sequences of the sixty-five LAB isolates. The type strains *Lactiplantibacillus plantarum* ATCC 14917 and *Apilactobacillus kunkeei* ATCC 700308 have been used as references. The analysis was conducted with the MEGA X [85] program using the maximum likelihood method and the Kimura 2-parameter model [89]. The scale bar represents a 0.5% nucleotide sequence difference.

The partial 16S rRNA gene sequences of these bacteria were determined and compared with related bacteria in GenBank (http://www.ncbi.nlm.nih.gov/BLAST/, accessed on 2 January 2022). Sequence matches that showed the highest identity scores (99% and above) were considered acceptable for taxonomic placement at the species level. According to the 16S rRNA gene sequences, all isolated strains were used to construct a phylogenetic tree (Figure 1) using the MEGA X program [85] via the maximum likelihood method and Kimura 2-parameter model [89]. 

The phylogenetic analysis showed that the bacterial strains considered in this study belonged to 4 genera: *Apilactobacillus, Lactiplantibacillus, Fructobacillus* and *Enterococcus.* The predominant species were *A. kunkeei* (28 strains) and *L. plantarum* (24 strains)*,* followed by *Fructobacillus fructosus (7 strains), Enterococcus faecalis (3 strains), Fructobacillus ficulneus* (1 strain) and *Fructobacillus pseudoficulneus* (1 strain).

### 3.2. Antimicrobial Activity 

The antagonistic activity of sixty-five LABs against *P. larvae* ATCC 9545 and *M. plutonius* ATCC 35311 was investigated. Four *L. plantarum* (LP 31, LP 42, LP 148 and LP 179) and four *A. kunkeei* (ALK 181, ALK 222, ALK 268 and ALK 385) strains demonstrated the greatest antimicrobial activity against the two pathogens (ZOI > 4 mm) and were selected for a subsequent agar well diffusion test using the CFS of the LAB cultures. Table S3 (Supplementary Materials) shows the list of selected LABs with the corresponding sequences and GenBank accession numbers.

CFS matrices, obtained from the *L. plantarum* and *A. kunkeei* strains, caused the growth inhibition of both pathogens. The numeric data of the antimicrobial activity (mm ZOI) are reported in Table 1 and highlight significant strain-specific differences, not species-related differences.

The ZOI values of the CFSs against *P. larvae* ranged from 13.7 mm to 16.3 mm. The lowest inhibition values were recorded for strains LP 179 and ALK 385 while the highest value was recorded for the CFS of the strain ALK 222. In the tests against *M. plutonius*, ZOI ranged from a minimum of 12.0 mm (LP 179) to a maximum of 16.0 mm (LP 148). 

### 3.3. Hydrophobicity and Auto-Aggregation

**Figure 2:**
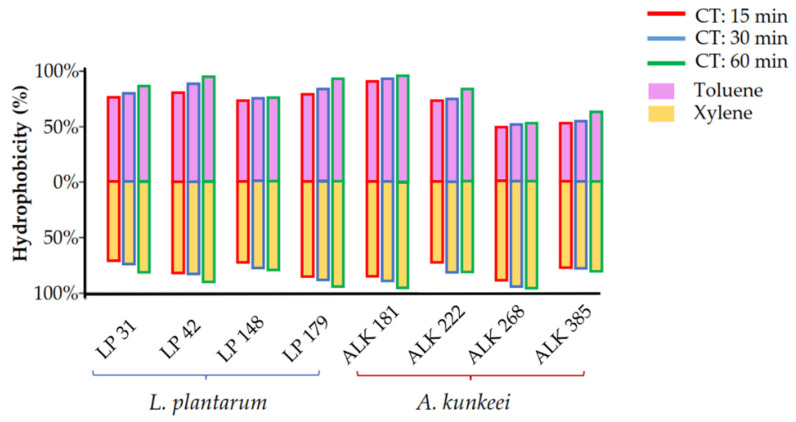
Adhesion of the *Lactiplantibacillus plantarum* and *Apilactobacillus kunkeei* strains to toluene and xylene expressed as hydrophobicity (%) after different contact times (CT; 15, 30, and 60 min).

The results obtained in the BATH assay are graphically reported in Figure 2 and numerically in Table 2. Adhesion to the two hydrocarbons gradually increased during the duration of the test. After 60 min, *L. plantarum* LP 42, LP 179 and *A. kunkeei* ALK 181 showed an adhesion to xylene and toluene greater than 90%. *A. kunkeei* ALK 268 exhibited particular behaviour, as it already showed the highest adhesion to xylene (>96.62%) and the lowest adhesion to toluene (47.34%) after 15 min. The overall data showed similar behaviour among the two tested bacterial species.

**Figure 3:**
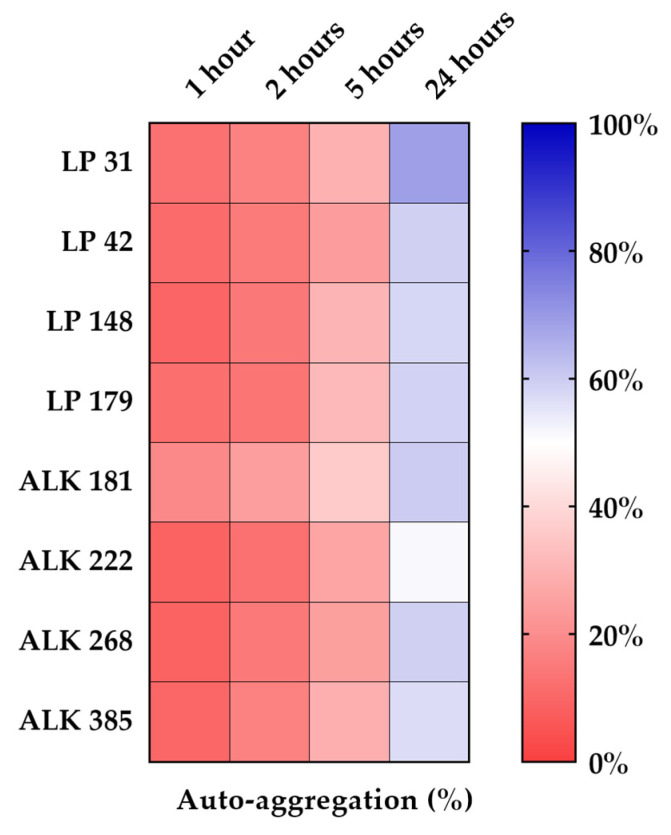
Percentage of auto-aggregation (AA%) of the *Lactiplantibacillus plantarum* and *Apilactobacillus kunkeei* strains.

In Figure 3, the heat map depicts the results of the auto-aggregation tests. The numeric data are reported in Table 3. The eight LAB strains showed progressive aggregation over time with often significant differences among them. The auto-aggregation values after 2 h had a similar trend for the two LAB species. After 5 and 24 h, significant differences between strains were observed. After 1, 2, and 5 h, ALK 181 strain had the highest percentage of auto-aggregation (19.0%, 24.40% and 36.1%, respectively). After 24 h, all strains showed aggregation values above 50%. The best performing strain was LP 31 (68.8%), while the lowest value was recorded for strain ALK 222 (51.1%).

### 3.4. Biochemical Characterization

The carbohydrate assimilation patterns, detected using the API 50 CHL kit, are presented in Table S4 (Supplementary Materials). Unlike the *A. kunkeei* strains, the four *L. plantarum* strains were able to assimilate raffinose, while only the *A. kunkeei* strains were capable of assimilating 5-keto-gluconate. *L. plantarum* LP 179 and *A. kunkeei* ALK 268 were the only tested bacteria capable of assimilating the rhamnose. *L.* *plantarum* LP 31 was the only tested bacterium capable of assimilating the pentose sugar L-xylose. The disaccharides gentiobiose and turanose were assimilated by the four *A. kunkeei* strains. Gentiobiose was also assimilated by *L. plantarum* LP 31 and LP 42, while *L. plantarum* LP 42 and LP 148 were capable of assimilating turanose. Gluconate was only assimilated by *L. plantarum* LP 31 and LP 179 strains. All LP strains assimilated sorbose, methyl-α-D-mannopyranoside and methyl-α-D-glucopyranoside. Moreover, the strain LP 31 was able to assimilate most of the tested carbohydrates.

Enzymatic profiles were detected with the API ZYM kit and are shown in Table S5 (Supplementary Materials). The four *A. kunkeei* exhibited very similar enzyme profiles. The differences among the ALK isolates were recorded regarding α-galactosidase, β-glucosidase and β-glucuronidase activities; however, *A. kunkeei* ALK 181 and ALK 268, showed no β-glucuronidase activity. The four *A. kunkeei* strains did not produce lipase, trypsin, α-chymotrypsin and α-mannosidase.

With regards to the *L. plantarum* enzymatic profile, the four tested strains showed N-acetyl- β-glucosaminidase, α-glucosidase, β-galactosidase and β-glucosidase activities. Only the LP 31 strain did not possess the alkaline phosphatase, α-galactosidase and esterase enzymes. Moreover, L42 and L179 strains were negative for esterase-lipase, leucine and valine arylamidase activities. The four *L. plantarum* strains did not produce the following enzymes: acid phosphatase, cystine arylamidase, lipase, trypsin, naphthol-AS-BI-phosphohydrolase, α-chymotrypsin, α-fucosidase, α-mannosidase and β-glucuronidase.

## 4. Discussion

Our investigation of the LAB community in the honey bee gut showed that *A. kunkeei* and *L. plantarum* were the most numerically representative species. *A. kunkeei* is a highly versatile bacterium and can be found in fructose-rich niches, including honey, beebread, flowers and the gastrointestinal tract of honey bees [53,62,90,91]. *L. plantarum* is also a LAB usually isolated from the honey bee gut [92,93] and, as highlighted in one of our previous studies, is sometimes numerically very representative [60]. This confirms the extreme adaptability of this species to different environmental niches [71,94], including those rich in fructose [95]. 

LABs’ antimicrobial activity is due to several factors: nutritional competitiveness and the production of compounds such as organic acids, fatty acids, protein compounds, phenolic acids and hydrogen peroxide [96]. In this investigation, the inhibition test was performed by utilizing the CFS of LAB cultures by the agar well diffusion method. The eight LAB strains showed inhibitory activity against the vegetative growth of *P. larvae* ATCC 9545 and *M. plutonius* ATCC 35311. Our results show the absence of inhibition in the control test with MRS pH 3.8, suggesting that the antagonistic action is not related to organic acids but to the presence in CFS of other metabolites capable of inhibiting the two pathogens. Accordingly, further studies are required to determine the exact nature of these antimicrobial compounds and to assess their mechanism of action against *P. larvae* and *M. plutonius.* It is also important to test the antigerminative efficacy of these compounds against *P. larvae* spores.

A balanced gut microbiota, in addition to playing an antagonistic role against pathogens, can also be involved with its metabolic activities in the digestive process of bees [11,97]. In our analysis of enzyme activity profiles, determined using the API-ZYM test, the eight strains of *L. plantarum* and *A. kunkeei* were found to possess glycosidase activity. Beta-glycosidase is important because, in combination with other enzymes, including cellulase and hemicellulose produced by honey bee gut symbionts such as *Gilliamella*, it contributes to the hydrolysis of cellulose [98]. Alpha-glycosidase is an enzyme that can hydrolyze maltose to glucose and is also directly involved in the degradation of starch granules [99,100]. Several carbohydrates present in the honey bee diet are toxic because these insects do not possess functional enzymes to metabolize them [101]. The results obtained in the carbohydrate assimilation test showed that the four *L. plantarum* strains are capable of metabolizing the monosaccharides L-arabinose, galactose, mannose, and the oligosaccharides melibiose, lactose, melezitose and raffinose. The four *A. kunkeei* assimilated L-arabinose, galactose, mannose, melibiose, lactose and melezitose. *L. plantarum* LP 179 and *A. kunkeei* ALK 268 were also able to metabolize rhamnose and *L. plantarum* LP 31 was the only bacterium able to assimilate L-xylose.

All of the above-mentioned carbohydrates are considered to be potentially toxic to the honey bee and may be contained in traces in the natural nectar derived from the hydrolysis of pectin, or synthesized as melezitose [101]. This sugar is a trisaccharide, composed of monosaccharide glucose and disaccharide turanose. It can be produced by aphids and is the main carbohydrate contained in honeydew [98,102,103]. The use and the role of the selected LAB strains as probiotics, thanks to their specific enzymatic activities, can contribute to the breakdown of complex polysaccharides and metabolize toxic sugars and consequently improve the dietary tolerance of honey bees [99].

The ability of probiotics to adhere to the intestinal *epithelium* is an important prerequisite for biofilm formation in the host intestinal tract. This ability, along with their antimicrobial activity are important features that can hinder the colonization of undesirable microorganisms [86,104,105]. The gut adherence ability of probiotic bacteria involves different types of surface properties, including hydrophobicity and auto-aggregation [106,107,108]. Auto-aggregation mechanisms generally involve various molecules including cell surface proteins, exopolysaccharides, carbohydrates, glycoproteins, teichoic and lipoteichoic acids [109]. The eight LAB strains were observed to have a high level of autoaggregation, which is a recommended characteristic for a good probiotic strain [110]. The adherence capacity is a strain-specific property due to several interactions between hydrophobic and hydrophilic components of the cell bacterial surface [106]. In our tests, the hydrophobicity was evaluated by the BATH method using xylene and toluene as hydrocarbons. The results showed that the LAB strains had high values of hydrophobicity and were in line with previous investigations [86]. Thanks to their cell surface properties, these LABs used as probiotic supplements in honey bees’ diets can persist in the intestinal tract where, especially during foraging, there is an intense flow of water and nectar.

The virulent action of *P. larvae* and *M. plutonius* is based on a few key steps: growth dynamics; attachment to host cells by the production of biologically active compounds, such as adhesins; and the production of enzymes degrading the peritrophic matrix, enabling the pathogens to directly attack the epithelial cells [111,112,113,114].

Probiotic bacteria, by binding to receptors in the intestinal mucosa, can inhibit the adherence of pathogenic microorganisms that are subsequently eliminated from the intestine.

## 5. Conclusions

In our scientific investigation, the evaluation of the functional properties of the eight strains of *A. kunkeei* and *L. plantarum* was carried out in vitro and therefore the good probiotic potential highlighted does not axiomatically result in health benefits for the honey bee colonies. Therefore, further investigations are needed to evaluate, in vivo orin situ, the role that these LABs, used as dietary supplements, can play in safeguarding and improving honey bee health. In particular, it is necessary to evaluate the contribution that these bacteria can make to the biocontrol strategy against EFB and AFB diseases.

## Figures and Tables

**Table 1:** Antimicrobial activity by cell-free supernatant (CFS) of the tested *Lactiplantibacillus plantarum* (LP) and *Apilactobacillus kunkeei* (ALK) strains against *Paenibacillus larvae* and *Melissococcus plutonius*. The data (mean ± SD; *n* = 3) were expressed as zone of inhibition-ZOI (mm). Different lowercase letters (a–d) in each row indicate significant differences (*p* < 0.05).

	Bacterial Strains							
Matrices (CSF)	LP31	LP42	LP148	LP179	ALK181	ALK222	ALK268	ALK385
*P. larvae*	14.9 ± 0.2 ^b^	15.1 ± 0.5 ^b^	15.8 ± 0.3 ^a^	13.9 ± 0.4 ^c^	15.5 ± 0.5 ^a^	16.3 ± 0.2 ^a^	15.0 ± 0.4 ^b^	13.7 ± 0.2 ^c^
*M. plutonius*	15.1 ± 0.3 ^a^	15.1 ± 0.3 ^a^	16.0 ± 0.1 ^a^	12.0 ± 0.2 ^d^	15.9 ± 0.4 ^a^	14.0 ± 0.3 ^b^	14.2 ± 0.3 ^b^	13.0 ± 0.5 ^c^

**Table 2:** Adhesion to toluene and xylene of the *Lactiplantibacillus plantarum* and *Apilactobacillus kunkeei* tested strains (LP 31, LP 42, LP 148, LP 179, ALK 181, ALK 222, ALK 268 and ALK 385) after different contact times. The data (mean ± SD; *n* = 3) were expressed as percentage of hydrophobicity, different lowercase letters in each row and uppercase letters in each column indicate significant differences (*p* < 0.05).

	**Hydrophobicity (%) Xylene**							
**Time**	**LP31**	**LP42**	**LP148**	**LP179**	**ALK181**	**ALK222**	**ALK268**	**ALK385**
15	70.8 ± 1.3 ^Cc^	80.5 ± 2.6 ^Bb^	72.2 ± 1.8 ^Bc^	85.3 ± 1.1 ^Bb^	84.0 ± 1.7 ^Bb^	72.3 ± 2.7 ^Bc^	95.4 ± 1.5 ^Aa^	76.1 ± 2.6 ^Ac^
30	76.0 ± 2.2 ^Bc^	81.4 ± 1.9 ^Bc^	77.9 ± 1.7 ^Ac^	87.8 ± 2.9 ^Ab^	88.6 ± 1.8 ^Ab^	79.6 ± 2.6 ^Ac^	97.3 ± 1.5 ^Aa^	76.2 ± 1.0 ^Ac^
60	82.8 ± 3.9 ^Ac^	90.9 ± 2.3 ^Ab^	78.7 ± 1.2 ^Ac^	91.3 ± 1.4 ^Ab^	91.9 ± 1.9 ^Ab^	81.9 ± 3.3 ^Ac^	98.0 ± 0.5 ^Aa^	80.1 ± 2.3 ^Ac^
	**Hydrophobicity (%) Toluene**							
**Time**	**LP31**	**LP42**	**LP148**	**LP179**	**ALK181**	**ALK222**	**ALK268**	**ALK385**
15	74.7 ± 2.0 ^Cc^	77.9 ± 0.5 ^Cb^	72.2 ± 1.7 ^Ac^	79.1 ± 0.8 ^Cb^	92.1 ± 1.6 ^Aa^	72.8 ± 0.4 ^Cc^	47.7 ± 1.0 ^Ae^	53.1 ± 0.4 ^Bd^
30	77.4 ± 2.5 ^Bd^	88.4 ± 0.4 ^Bb^	73.6 ± 1.2 ^Ae^	84.0 ± 0.5 ^Bc^	92.8 ± 0.3 ^Aa^	75.5 ± 1.2 ^Bd^	47.8 ± 1.9 ^Ag^	54.9 ± 2.3 ^Bf^
60	85.5 ± 2.4 ^Ab^	94.1 ± 0.9 ^Aa^	74.4 ± 0.4 ^Ac^	93.5 ± 0.7 ^Aa^	93.2 ± 0.3 ^Aa^	83.1 ± 1.7 ^Ab^	50.0 ± 0.3 ^Ae^	60.2 ± 1.2 ^Ad^

**Table 3:** Auto-aggregation (AA) at 35 °C of the tested lactic acid bacteria (*Lactiplantibacillus plantarum*: LP 31, LP 42, LP 148 and LP 179; *Apilactobacillus kunkeei*: ALK 181, ALK 222, ALK 268 and ALK 385). The data (mean ± SD; *n* = 3) were expressed as percentage of AA, different lowercase letters in each row and uppercase letters in each column indicate significant differences (*p* < 0.05).

	Auto-Aggregation %							
Time (h)	LP31	LP42	LP148	LP179	ALK181	ALK222	ALK268	ALK385
1	12.4 ± 0.3 ^Db^	11.2 ± 0.5 ^Cb^	9.5 ± 0.5 ^Db^	12.3 ± 0.9 ^Cb^	19.0 ± 0.9 ^Da^	8.93 ± 0.2 ^Cb^	9.1 ± 0.1 ^Db^	10.2 ± 0.4 ^Db^
2	17.0 ± 1.3 ^Cb^	15.1 ± 1.8 ^Cb^	14.5 ± 1.6 ^Cb^	13.8 ± 1.2 ^Cb^	24.4 ± 1.9 ^Ca^	12.4 ± 0.7 ^Cb^	14.7 ± 0.8 ^Cb^	17.0 ± 1.6 ^Cb^
5	29.4 ± 3.0 ^Bb^	24.0 ± 2.6 ^Bc^	30.2 ± 2.5 ^Bb^	31.5 ± 1.8 ^Ba^	36.1 ± 2.4 ^Ba^	26.3 ± 1.7 ^Bb^	24.8 ± 1.0 ^Bc^	28.8 ± 2.1 ^Bb^
24	68.8 ± 3.6 ^Aa^	59.2 ± 2.5 ^Ab^	57.9 ± 2.0 ^Ab^	58.7 ± 2.9 ^Ab^	60.0 ± 2.3 ^Ab^	51.1 ± 2.2 ^Ac^	59.2 ± 1.7 ^Ab^	56.8 ± 2.4 ^Ab^

## Data Availability

The data presented in this study are available in the Supplementary Materials.

## Supplementary Materials

The following supporting information can be downloaded at: https://www.mdpi.com/article/10.3390/vetsci9050236/s1, Table S1: Screening of antimicrobial activity; Table S2: API 50 medium; Table S3: List of selected LABs; Table S4: Carbohydrate assimilation profiles; Table S5: Enzymatic profiles.
