# Supplementary material for: Antimicrobial Activity from Putative Probiotic Lactic Acid Bacteria for the Biological Control of American and European Foulbrood Diseases
Source: Vet Sci. 2022 May 12;9(5):236. doi: 10.3390/vetsci9050236 (PMC9143654; doi:10.3390/vetsci9050236)
Supplement: Supplementary file 1 [file vetsci-09-00236-s001.zip › Table S1 Screening of antimicrobial activity.pdf]

Table S1. Screening of antimicrobial activity of the sixty-five LAB strains performed by spot agar assay.

CFS	*P. larvae*	*M. plutonius*	*CFS*	*P. larvae*	*M. plutonius*
Apilactobacillus kukeii 196	1.10±0.10	0.66±0.05	*Fructobacillus ficulneus 453*	1.16±0.15	0.33±0.05
Apilactobacillus kukeii 198	2.03±0.15	1.06±0.22	*Fructobacillus fructosus 267*	0.26±0.05	0.96±0.11
Apilactobacillus kukeii 210	0.70±0.11	1.33±0.15	*Fructobacillus fructosus 294*	0.56±0.11	1.51±0.23
Apilactobacillus kunkeei 129	3.00±0.17	1.96±0.12	*Fructobacillus fructosus 295*	1.03±0.15	1.56±0.11
Apilactobacillus kunkeei 15	3.23±0.15	1.63±0.11	*Fructobacillus fructosus 352*	0.82±0.10	2.73±0.15
Apilactobacillus kunkeei 172	2.21±0.24	2.16±0.26	*Fructobacillus fructosus 353*	1.93±0.11	1.86±0.15
Apilactobacillus kunkeei 174	2.1±0.12	1.14±0.14	*Fructobacillus fructosus 360*	2.94±0.14	2.56±0.11
Apilactobacillus kunkeei 176	1.64±0.22	3.16±0.27	*Fructobacillus pseudoficulneus 455*	0.83±0.05	1.43±0.15
Apilactobacillus kunkeei 177	0.54±0.13	2.86±0.15	*Lactiplantibacillus plantarum 108*	1.46±0.15	0.83±0.05
Apilactobacillus kunkeei 180	3.33±0.25	1.89±0.17	*Lactiplantibacillus plantarum 110*	2.74±0.17	1.86±0.15
Apilactobacillus kunkeei 193	1.46±0.25	2.66±0.15	*Lactiplantibacillus plantarum 122*	3.16±0.15	2.36±0.15
Apilactobacillus kunkeei 251	2.76±0.11	1.31±0.12	*Lactiplantibacillus plantarum 129*	3.16±0.26	1.26±0.05
Apilactobacillus kunkeei 26	1.25±0.17	1.26±0.15	*Lactiplantibacillus plantarum 130*	2.53±0.15	2.46±0.15
Apilactobacillus kunkeei 298	0.56±0.15	1.25±0.17	*Lactiplantibacillus plantarum 131*	1.43±0.22	2.96±0.25
Apilactobacillus kunkeei 299	3.43±0.42	2.06±0.11	*Lactiplantibacillus plantarum 257*	2.53±0.40	1.41±0.17
Apilactobacillus kunkeei 30	2.76±0.15	2.56±0.05	*Lactiplantibacillus plantarum 3*	0.96±0.15	2.43±0.32
Apilactobacillus kunkeei 300	1.40±0.20	3.03±0.15	*Lactiplantibacillus plantarum 53*	1.82±0.17	3.28±0.26
Apilactobacillus kunkeei 314	3.03±0.15	1.96±0.15	*Lactiplantibacillus plantarum 82*	0.97±0.14	2.76±0.22
Apilactobacillus kunkeei 32	0.36±0.11	2.76±0.25	*Lactiplantibacillus plantarum 83*	2.46±0.22	2.23±0.25
Apilactobacillus kunkeei 328	0.16±0.05	2.90±0.13	*Lactiplantibacillus plantarum 84*	1.53±0.20	1.19±0.17
Apilactobacillus kunkeei 338	2.06±0.25	1.23±0.15	*Lactiplantibacillus plantarum 86*	1.06±0.15	0.33±0.05
Apilactobacillus kunkeei 467	0.97±0.11	1.36±0.11	*Lactiplantibacillus plantarum 92*	2.16±0.14	0.66±0.15
Apilactobacillus kunkeei 78	1.53±0.35	2.92±0.26	*Lactiplantibacillus plantarum ATCC 14917*	1.16±0.15	1.83±0.11
Apilactobacillus kunkeei 99	1.23±0.05	2.26±0.21	*Lactiplantibacillus plantarum LP100*	2.13±0.15	0.56±0.15
Apilactobacillus kunkeei ALK181	5.83±0.36	6.72±0.25	*Lactiplantibacillus plantarum LP148*	7.96±0.25	6.93±0.32
Apilactobacillus kunkeei ALK222	6.00±0.35	5.63±0.22	*Lactiplantibacillus plantarum LP179*	5.91±0.36	5.51±0.37
Apilactobacillus kunkeei ALK268	5.13±0.23	6.16±0.21	*Lactiplantibacillus plantarum LP212*	2.40±0.25	1.16±0.15
Apilactobacillus kunkeei ALK385	6.43±0.35	5.63±0.32	*Lactiplantibacillus plantarum LP25*	0.86±0.05	0.53±0.05
Apilactobacillus kunkeei ATCC 700308	1.03±0.15	1.46±0.11	*Lactiplantibacillus plantarum LP31*	7.46±0.25	5.96±0.25
Enterococcus faecalis 145	0.67±0.14	1.36±0.15	*Lactiplantibacillus plantarum LP42*	8.12±0.37	4.93±0.32
Enterococcus faecalis 146	1.31±0.22	2.3±0.09	*Lactiplantibacillus plantarum LP8*	1.33±0.15	2.00±0.34
Enterococcus faecalis 390	2.13±0.15	3.26±0.34	*Lactiplantibacillus plantarum LP86*	2.63±0.37	1.10±0.17
			Lactiplantibacillus plantarum LP95	1.83±0.15	2.56±0.15
