# Supplementary material for: Antimicrobial Activity from Putative Probiotic Lactic Acid Bacteria for the Biological Control of American and European Foulbrood Diseases
Source: Vet Sci. 2022 May 12;9(5):236. doi: 10.3390/vetsci9050236 (PMC9143654; doi:10.3390/vetsci9050236)
Supplement: Supplementary file 1 [file vetsci-09-00236-s001.zip › Table S2 API 50 medium.pdf]

Table S2. Composition of API 50 medium.

**API 50 CHL Medium composition**	Polpeptone (bovine/porcine origin)	10 g
	Yeast extract	5 g
	Tween 80	1 mL
	Dipotassium phosphate	2 g
	Sodium acetate	5 g
	Diammonium citrate	2 g
	Magnesium sulfate	0.20 g
	Manganese sulfate	0.05 g
	Bromcresol purple	0.17 g
	Demineralized water	1000 mL
	pH: 6.7-7.1	
