# Supplementary material for: Antimicrobial Activity from Putative Probiotic Lactic Acid Bacteria for the Biological Control of American and European Foulbrood Diseases
Source: Vet Sci. 2022 May 12;9(5):236. doi: 10.3390/vetsci9050236 (PMC9143654; doi:10.3390/vetsci9050236)
Supplement: Supplementary file 1 [file vetsci-09-00236-s001.zip › Table S4 Carbohydrate assimilation profiles.pdf]

Table S4. Carbohydrate assimilation profiles of the selected *Lactiplantibacillus plantarum* and *Apilactobacillus kunkeei* strains using API 50 CHL test kit (+ positive; − negative).

Carbohydrate	*Lp. plantarum* strains				*Al. kunkeei* strains			
	**LP 31**	**LP 42**	**LP 148**	**LP 179**	**ALK 181**	**ALK 222**	**ALK 268**	**ALK 385**
Glycerol	-	-	-	-	-	-	-	-
Erythriol	-	-	-	-	-	-	-	-
D-Arabinose	-	-	-	-	-	-	-	-
L-Arabinose	+	+	+	+	+	+	+	+
Ribose	+	+	+	+	+	+	+	+
D-Xylose	-	-	-	-	-	-	-	-
L-Xylose	+	-	-	-	-	-	-	-
Adonitol	-	-	-	-	-	-	-	-
Methyl-β-D-Xylopyranoside	-	-	-	-	-	-	-	-
Galactose	+	+	+	+	+	+	+	+
Glucose	+	+	+	+	+	+	+	+
Fructose	+	+	+	+	+	+	+	+
Mannose	+	+	+	+	+	+	+	+
Sorbose	+	+	+	+	-	-	-	-
Rhamnose	-	-	-	+	-	-	+	-
Dulcitol	-	-	-	-	-	-	-	-
Inositol	-	-	-	-	-	-	-	-
Mannitol	+	+	+	+	+	+	+	+
Sorbitol	+	+	+	+	+	+	+	+
Methyl-α-D-Mannopyranoside	+	+	+	+	-	-	-	-
Methyl-α-D-Glucopyranoside	+	+	+	+	-	-	-	-
N-Acetylglucosamine	+	+	+	+	+	+	+	+
Amygdalin	+	+	+	+	+	+	+	+
Arbutin	+	+	+	+	+	+	+	+
Esculin	+	+	+	+	+	+	+	+
Salicin	+	+	+	+	+	+	+	+
Cellobiose	+	+	+	+	+	+	+	+
Maltose	+	+	+	+	+	+	+	+
Lactose	+	+	+	+	+	+	+	+
Melibiose	+	+	+	+	+	+	+	+
Sucrose	+	+	+	+	+	+	+	+
Trehalose	+	+	+	+	+	+	+	+
Inulin	-	-	-	-	-	-	-	-
Melezitose	+	+	+	+	+	+	+	+
Raffinose	+	+	+	+	-	-	-	-
Starch	-	-	-	-	-	-	-	-
Glycogen	-	-	-	-	-	-	-	-
Xylitol	-	-	-	-	-	-	-	-
Gentiobiose	+	+	-	-	+	+	+	+
Turanose	-	+	+	-	+	+	+	+
Lyxose	-	-	-	-	-	-	-	-
Tagatose	-	-	-	-	-	-	-	-
D-Fucose	-	-	-	-	-	-	-	-
L-Fucose	-	-	-	-	-	-	-	-
D-Arabitol	-	-	-	-	-	-	-	-
L-Arabitol	-	-	-	-	-	-	-	-
Gluconate	+	-	-	+	-	-	-	-
2-keto-gluconate	-	-	-	-	-	-	-	-
5-keto-gluconate	-	-	-	-	+	+	+	+
