# Supplementary material for: Antimicrobial Activity from Putative Probiotic Lactic Acid Bacteria for the Biological Control of American and European Foulbrood Diseases
Source: Vet Sci. 2022 May 12;9(5):236. doi: 10.3390/vetsci9050236 (PMC9143654; doi:10.3390/vetsci9050236)
Supplement: Supplementary file 1 [file vetsci-09-00236-s001.zip › Table S5 Enzymatic profiles.pdf]

Table S5. Enzymatic profiles of the 8 tested LAB strains using the API-ZYM system (+ positive; - negative).

Enzyme	*Lp. plantarum* strains				*Al. kumkeei* strains			
	**LP 31**	**LP 42**	**LP 148**	**LP 179**	**ALK 181**	**ALK 222**	**ALK 268**	**ALK 385**
Acid phosphatase	-	-	-	-	+	+	+	+
Alkaline phosphatase	-	+	+	+	+	+	+	+
Cystine arylamidase	-	-	-	-	+	+	+	+
Esterase (C4)	-	+	+	+	+	+	+	+
Esterase lipase (C8)	+	-	+	-	+	+	+	+
Leucine arylamidase	+	-	+	-	+	+	+	+
Lipase (C14)	-	-	-	-	-	-	-	-
N-acetyl-b-glucosaminidase	+	+	+	+	+	+	+	+
Naphthol-AS-BI-phosphohydrolase	-	-	-	-	+	+	+	+
Trypsin	-	-	-	-	-	-	-	-
Valine arylamidase	+	-	+	-	+	+	+	+
α-Chymotrypsin	-	-	-	-	-	-	-	-
α-Fucosidase	-	-	-	-	+	+	+	+
α -Galactosidase	-	+	+	+	-	+	-	-
α-Glucosidase	+	+	+	+	+	+	+	+
α-Mannosidase	-	-	-	-	-	-	-	-
β-Galactosidase	+	+	+	+	+	+	+	+
β -Glucosidase	+	+	+	+	+	+	-	+
β-Glucuronidase	-	-	-	-	-	+	-	+
